# P-1427. Continuation of HIV and hepatitis C care among refugees migrating from Ukraine - Five Times Ninety project

**DOI:** 10.1093/ofid/ofae631.1602

**Published:** 2025-01-29

**Authors:** Justyna Kowalska, Sergii Antoniak, Olena Samsonova

**Affiliations:** Medical University of Warsaw, Warsaw, Mazowieckie, Poland; Gromashevsky Institute of epidemiology and infectious diseases within NAMS of Ukraine, Kyiv, Kyyivs'ka Oblast', Ukraine; Public Health Center of the Ministry of Health of Ukraine, Kyiv, Kyyivs'ka Oblast', Ukraine

## Abstract

**Background:**

Five Times Ninety project investigates linkage to care among war refugees living with HIV displaced from Ukraine to Poland.

**Figure 1.**
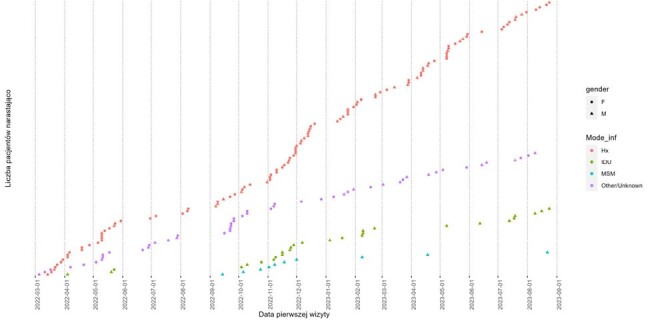
Gender and mode of HIV infection of Ukrainian refugees stratified by time of registering to care in Warsaw

**Methods:**

Refugees who registered to HIV care in Warsaw were followed according to “Standardized protocol for clinical management and medical data sharing for PLHIV among refugees from Ukraine” developed by WHO/CHIP,EACS, ECEE and ECDC (https://iris.who.int/handle/10665/353083). For each patient who signed informed request data were linked to database at the Public Health Center (PHC) Ministry of Health Ukraine, and sent back on standardized form.

**Figure 2.**
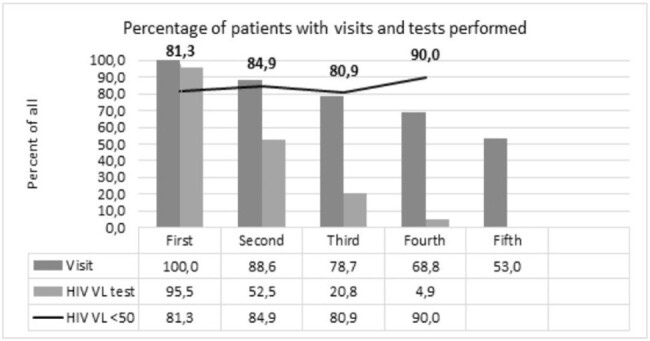
Cascade of HIV care of Ukrainian refugees registered to care in Warsaw

**Results:**

Among 205 refugees who registered to HIV care in Warsaw (5 Mar2022 - 31Aug2023) 202 (98.7%) were linked with PHC database, 121 (59.9%) were female, median age 40.0 (range 18-69, IQR 36.0-46.0), migrating from Central (67; 33.2%), South (65; 32.2%), East (39; 19.3%), West (27; 13.4%) and North (4; 2.0%) Ukraine. Mode of HIV infection was heterosexual in 113 (55.9%), other/unknown in 51 (25.2%), injecting drug use in 28 (13.9%) and MSM in 10 (4.9%) persons. Women and persons who acquired HIV through heterosexual contacts were more likely to migrate earlier, but age structure did not significantly change over time (Figure 1). On 31Dec2023 in total 128 (63.4%) patients remained in care in Warsaw.

189 patients (93.1%) had HIV viral load measured both in Ukraine and Poland, 173 (91.5%) and 155 (82.0%) were undetectable, respectively. This translates into 9.5% loss of ART efficacy during a transition period. These improved over time (Figure 1). Switching of antiretroviral therapy occurred often, yet majority remained on integrase inhibitor (194; 98.0%) and tenofovir (176; 88.9%), Figure 4.

148 persons (73.6%) were tested with HCV serology in Ukraine and 187 (93.0%) in Poland. Of 59 (31.5%) tested positive in Poland, 24 were post-DAA therapy in Ukraine. Of 35 who were tested with PCR in Poland, 10 had undetectable HCV RNA, 25 had detectable HCV RNA of whom 10 already went through DAA therapy, Figure 3.

**Figure 3.**
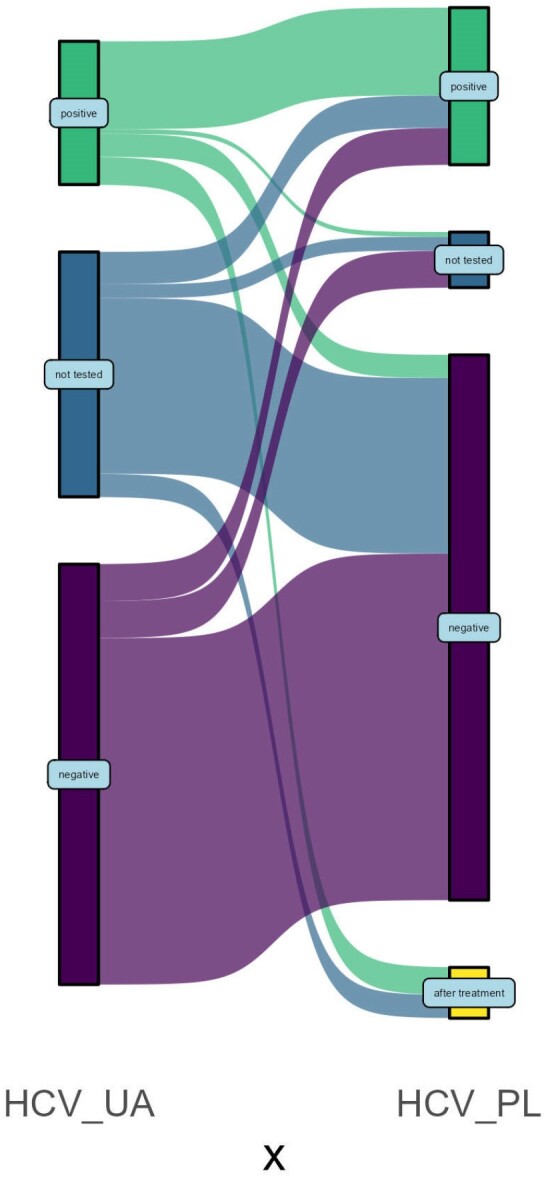
HCV testing and treatment for Ukrainian refugees registered to care in Warsaw

**Conclusion:**

Standardized protocol proved to be an efficient for medical data exchange. Viral suppression among Ukrainian refugees on ART decreased slightly, but in general remained high and improved with time. Routine HCV screening is a necessity, especially because there are no restrains to DAA treatment access in Poland.

**Figure 4.**
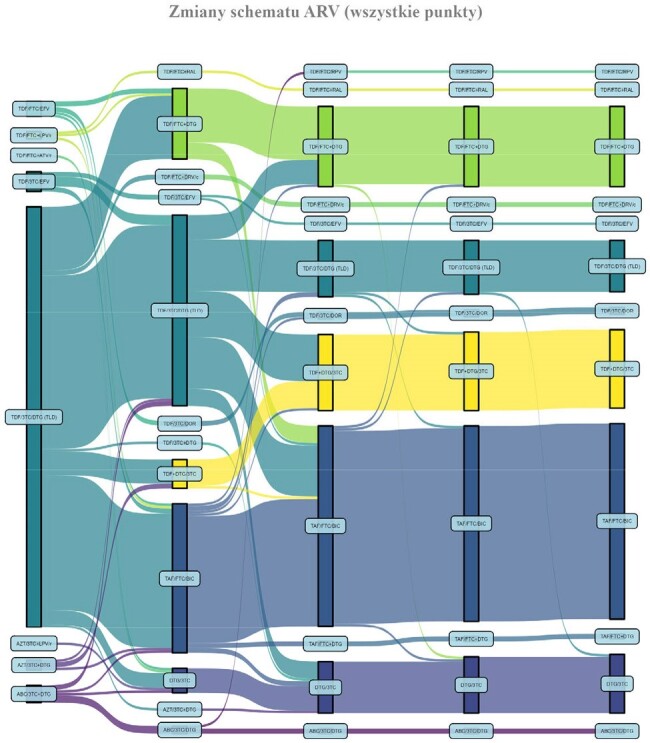
Antiretroviral therapy switches among refugees registered to care in Warsaw

**Disclosures:**

**All Authors**: No reported disclosures

